# Neuron-specific enolase during the therapy in patients with alcohol use disorder and mood disorders

**DOI:** 10.1192/j.eurpsy.2021.1104

**Published:** 2021-08-13

**Authors:** L. Levchuk, O. Roshchina, G. Simutkin, N. Bokhan, S. Ivanova

**Affiliations:** 1 Laboratory Of Molecular Genetics And Biochemistry, Mental Health Research Institute, Tomsk National Research Medical Center of the Russian Academy of Sciences, Tomsk, Russian Federation; 2 The Department Of Depressive States, Mental Health Research Institute, Tomsk National Research Medical Center of the Russian Academy of Sciences, Tomsk, Russian Federation; 3 The Department Of Addictive States, Mental Health Research Institute, Tomsk National Research Medical Center of the Russian Academy of Sciences, Tomsk, Russian Federation

**Keywords:** mood disorders, neuron-specific enolase, alcohol use disorder

## Abstract

**Introduction:**

Studies of the pathophysiology of mental disorders indicate the involvement of neurobiological processes, including the release of neurospecific proteins in biological substances.

**Objectives:**

The purpose of this study was to research the level of neuron-specific enolase in patients with alcohol use disorder and mood disorders during the therapy.

**Methods:**

The studied groups included patients with alcohol use disorder (AUD, F10.2, ICD-10; n=41), patients with mood disorders (MD, F32, F33, ICD-10; n=39), patients with co-morbidity of AUD and MD (n=31) and 20 healthy controls. Severity of depressive symptoms was assessed with HDRS-17 and CGI-S scales. The concentration of NSE were measured in serum by enzyme immunoassay. Рarticipants of the study were examined with clinical scales and laboratory analysis at baseline and on the 28th day of treatment. For statistical analysis we used the SPSS software.

**Results:**

The results of the study showed that all patients are characterized by an increased level of NSE (p>0.005 compared with control). Patients with AUD characterized by changes in the concentration of NSE during therapy (p>0.005 compared with patients after therapy). In patients with MD revealed correlation between the level of NSE on the 28th day of antidepressive therapy and the HDRS-17 score before treatment (r=0,421; p=0,018). In patients with co-morbidity correlation between the level of NSE and the CGI-S score before therapy was found (r=-0,537; p=0,001).

**Conclusions:**

The revealed correlations indicate the relationship between the severity of depressive symptoms and the level of NSE. Disclosure statement: This study was supported by the Russian Science Foundation, grant No. 19-15-00023.

**Conflict of interest:**

Disclosure statement: This study was supported by the Russian Science Foundation, grant No. 19-15-00023.

